# The prognostic significance of the treatment response of regional lymph nodes and the refinement of the current TNM staging system in locally advanced rectal cancer after neoadjuvant chemoradiotherapy

**DOI:** 10.1002/cam4.3553

**Published:** 2020-10-20

**Authors:** Jian Cui, Lin Zhang, Lin Yang, Yue‐Lu Zhu, Hui Fang, Bo Chen, Yi Ning, Hai‐Zeng Zhang

**Affiliations:** ^1^ Department of Colorectal Surgery and State Key Lab of Molecular Oncology National Cancer Center/National Clinical Research Center for Cancer/Cancer Hospital Chinese Academy of Medical Sciences and Peking Union Medical College Beijing China; ^2^ Department of Pathology National Cancer Center/National Clinical Research Center for Cancer/Cancer Hospital Chinese Academy of Medical Sciences and Peking Union Medical College Beijing China; ^3^ Department of Radiation Oncology National Cancer Center/National Clinical Research Center for Cancer/Cancer Hospital Chinese Academy of Medical Sciences and Peking Union Medical College Beijing China; ^4^ Meinian Public Health Institute Peking University Health Science Center Beijing China

**Keywords:** lymph node regression, neoadjuvant chemoradiotherapy, prognosis, rectal cancer, tumor regression grade

## Abstract

The current TNM staging system uses the same category definitions for both rectal cancer patients with and without neoadjuvant chemoradiotherapy (NCRT). However, ypTNM stage, especially ypN stage does not predict patient survival after NCRT well. Whether tumor regression in lymph nodes (LRG) may improve the prediction has not been well studied. In total, 358 patients with rectal cancer who received NCRT followed by radical resection were recruited from 2004 to 2015, and the median follow‐up time was 57.5 months. The main outcome measure was disease‐free survival (DFS). In univariate analysis, factors associated with DFS were ypT stage, ypN stage, number of negative lymph nodes (NLN), lymph node ratio (LNR), tumor regression grade (TRG), M‐TTRG (modified ypT stage by combining ypT stage and TRG), maximum LRG (LRGmax), sum score of LRG (LRGsum), LRG ratio (average value of LRGsum), and M‐NLRG (modified ypN stage by combining LRGmax and LNR). In the multivariate Cox regression analysis, M‐TTRG and M‐NLRG (*p* < 0.001 and *p* = 0.030, respectively) were significantly associated with DFS. The estimated 5‐year DFS rates were 86.6%, 60.3%, and 36.4% for patients with M‐NLRG‐0, M‐NLRG‐1, and M‐NLRG‐2, respectively (*p* < 0.001). A significant difference in survival was observed among patients with NCRT after incorporating TRG and LRG simultaneously into the current ypTNM staging system (*p* < 0.001). LRG was an important prognostic factor in rectal cancer patients treated with NCRT and could refine the ypTNM staging system. The modified ypTNM staging system in combination with LRGmax, LNR, and TRG could improve the DFS prediction in each subset of patients.

## Graphical abstract

Lymph node regression grade is an important prognostic factor in rectal cancer patients treated with neoadjuvant chemoradiotherapy and could refine the ypTNM staging system.

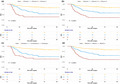

## 1 INTRODUCTION

Neoadjuvant chemoradiotherapy (NCRT) has now become the standard of care for locally advanced mid‐low rectal cancer.^1^ The American Joint Committee on Cancer (AJCC) 8^th^ TNM staging system has still been used to evaluate the prognosis of patients who received NCRT and employs the same category definitions as those for patients without NCRT. Some studies revealed that the current AJCC staging system could not assess prognosis precisely and was not a good predictor for patient survival after NCRT, especially in certain subgroups.^2^ NCRT induces a downstaging effect and various degrees of treatment response, ranging from no evidence of any treatment effect to pathological complete response (pCR) with no viable tumor identified.^3^, ^4^ The treatment response is evaluated by tumor regression grade (TRG) in primary tumor, which has been reported to have a significant impact on prognosis in a number of studies.^3^, ^5^, ^6^, ^7^ Thus, investigators suggested that the treatment response to NCRT should be included in the current AJCC TNM staging system.^8^ In a previous study, we developed a new classification metric, “M‐TTRG,” by combining the treatment response of the primary tumor and ypT, and the M‐TTRG could better predict long‐term survival.^8^

Lymph node metastasis is the most important prognostic factor in rectal cancer.^9^ Despite the downstaging effect of NCRT, lymph node status has been found to be significantly correlated with prognosis and clinical outcomes. NCRT leads to shrinkage of the primary tumor and metastatic tumors in regional lymph nodes.^10^ The treatment response of the lymph node is expected to be a more important prognostic factor than TRG. However, most studies on the prognostic value of tumor regression have focused on the primary tumor, and only a few studies have reported the histological effect of NCRT on regional lymph nodes.^11^, ^12^, ^13^ Caricato first analyzed the effect of chemoradiation on mesorectal lymph nodes in terms of tumor regression grade (tumor regression in lymph node or LRG) in rectal cancer. However, the long‐term prognostic outcome was not mentioned due to the small number of patients and the short follow‐up duration of the study.^12^ Another study enrolled 190 rectal cancer patients and showed that LRG was related to the recurrence of rectal cancer. However, the validation of the impact of validation of LRG on TNM stage and whether LRG could be included in the current staging system were not investigated in previous studies.^13^

In the current TNM staging system, ypN stage is categorized by the absolute number of positive lymph nodes (PLN): ypN0 (PLN = 0); ypN1 (PLN = 1–3); and ypN2 (PLN ≥4). Until now, the prognostic value of LRG has not been well studied or understood. NCRT can result in decreased lymph node retrieval, N downstaging effect and pN stage migration. Therefore, the current TNM staging system may not provide an accurate assessment of lymph node metastatic diseases. Moreover, several studies suggested that the number of negative lymph nodes and lymph node ratio (LNR) also had significant prognostic value.^14^, ^15^, ^16^ In this study, we evaluated the prognostic significance of LRG, and then, combined LRGmax and LNR into a new prognostic factor (M‐NLRG) that could predict disease‐free survival (DFS) better than ypN stage. Finally, we incorporated TRG and LRG into the current ypTNM staging system for cancer survival.

## 2 METHODS

### 2.1 Patients

A retrospective cohort study was carried out using an institutional database. Adult patients consecutively receiving NCRT and radical surgery for rectal cancer were selected for the study. Patients were enrolled retrospectively during the period from January 2004 to December 2015, were diagnosed with adenocarcinoma of the rectum, and underwent preoperative NCRT and surgery at Cancer Hospital, Chinese Academy of Medical Sciences. Initial staging was assessed by complete physical examination, digital rectal examination, colonoscopy, serum carcinoembryonic antigen (CEA), abdominal and pelvic spiral computed tomography (CT) scans, magnetic resonance imaging (MRI) or endorectal ultrasonography, chest radiographs/CT, and other radiologic evaluations according to individual patient characteristics.

Patients were considered for the study if they met the following criteria: (a) mid (6–10 cm from the anal verge) or lower (≤5 cm from the anal verge) primary rectal cancer; (b) locally advanced pathologically proven adenocarcinoma (cT3‐4 N0 or T_any_, N+); (c) NCRT followed by radical excision; and (d) no evidence of distant metastatic disease either before surgery or interoperation. Patients who had familial adenomatous polyposis, Lynch syndrome, synchronous, or metachronous second tumors were excluded. Patients without lymph nodes detected (ypNx) or with lateral lymph node involvement in the resected specimens and these patients who developed distant metastasis in 6 months after surgery were also excluded.

### 2.2 Treatment

Patients who received long‐course external beam chemoradiotherapy were given a median total dose of 50 Gy (40–50 Gy) delivered in 25 fractions. Concurrent chemotherapy was administered with a 5‐fluorouracil (5‐FU)‐based regimen with or without oxaliplatin for chemosensitization. Radical surgeries included low anterior resection (LAR), abdominoperineal resection (APR), or the Hartmann procedure according to the total mesorectal excision (TME) principle.

### 2.3 Tumor regression grade evaluation in the primary tumor and lymph nodes

Standard hematoxylin‐eosin and saffron staining of each paraffin block was performed for histological examination. All the slices were re‐evaluated by two professional gastrointestinal pathologists. TRG was evaluated according to the TRG system proposed by Mandard et al. in 1994.^6^ TRG was divided into five groups according to the following standards. TRG 1 was defined as complete regression; TRG 2 was defined as fibrosis with scattered tumor cells; TRG 3 was defined as fibrosis and tumor cells with a preponderance of fibrosis; TRG 4 was defined as fibrosis and tumor cells with a preponderance of tumor cells; and TRG 5 was defined as tumor tissue without any change of regression. As we previously reported, the M‐TTRG classification was a combination of the depth of tumor invasion (ypT stage) and the percentage of residual viable tumor (TRG) with weighting by β‐coefficients from multivariate analyses.^8^

### 2.4 LRG classifications

**FIGURE 1:**
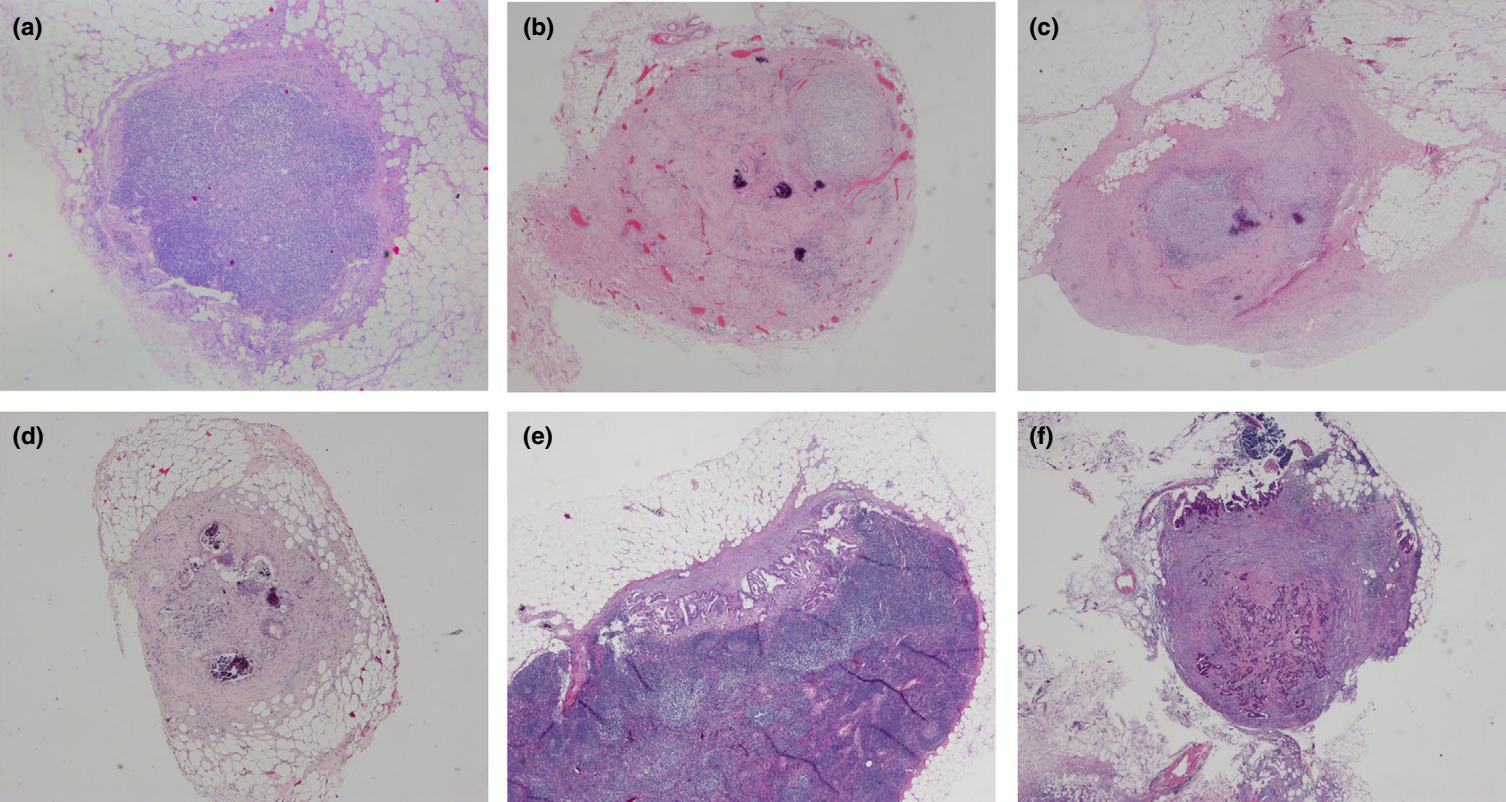
Representative lymph node regression grade (LRG). A, LRG0, negative lymph node. B, LRG1, complete regression with no residual tumor cells. C, LRG2, rare residual tumor cells. D, LRG3, fibrosis outgrown by residual tumor cells. E, LRG4, residual tumor cell outgrown by fibrosis. F, LRG5, absence of regression with no fibrosis

LRG of each lymph node was categorized by a change in the presence of tumor cells to the evidence of regression determined by the presence of fibrosis or mucous lakes with the same protocol as the primary tumor. LRG 1 was defined as complete regression with no residual tumor cells in lymph node. LRG2 was defined as rare residual tumor cells in lymph node. LRG3 was defined as fibrosis outgrown by residual tumor cells in lymph node. LRG4 was defined as residual tumor cell outgrown by fibrosis in lymph node. LRG5 was defined as the absence of regression with no fibrosis in lymph node. Though no residual metastatic tumors were detected, the true negative lymph nodes pre‐NCRT (with no fibrosis and tumor cells, LRG0) was distinguished from the complete response lymph nodes (with total fibrosis but no tumor cells, LRG1). Representative figures of each lymph node regression grade (LRG) are presented in Figure 1.

As each patient has a variable number of lymph nodes and each lymph node may exhibit a different regression grade based on its response to treatment, the maximum LRG (LRGmax), sum score of LRG (LRGsum) of each lymph node for an individual patient and LRG ratio (LRGsum divided by the number of PLN) were calculated.^13^ Patients with an LRGmax score of 0 were considered as all lymph nodes negative prior to NCRT. Patients with an LRGmax score of 1 were considered to have lymph nodes involvement prior to NCRT and achieved pathological complete response after NCRT.

To incorporate PLN, NLN, and LRG into one prognostic factor, LRGmax was multiplied by LNR to obtain a new parameter, M‐NLRG (modified number of lymph nodes and lymph node regression grade).

The X‐tile program was used to identify the optimal cut‐off value of LRGmax, LRGsum, LRG ratio, and M‐NLRG based on DFS.

### 2.5 Follow‐up

The patients were followed up and re‐examined routinely after surgery every 3 months for the first 2 years, every 6 months for the next 3 years, and then, yearly. Every half a year, all the patients were followed up through medical records, follow‐up letters and telephone class when necessary. The median follow‐up was 57.5 months (range from 9.6 to 148.1 months). The follow‐up time less than 9 month was defined as lost to follow‐up. The end point of the follow‐up was 1 April 2019.

### 2.6 Statistical analysis

Statistical analysis was performed using SPSS version 23.0 (SPSS Inc). Student's *t* test and univariate analysis were used for numeric data. The results are expressed as both the mean and median. DFS rates were calculated by using the Kaplan–Meier method. The potential prognostic factors for local recurrence or distant metastasis, screened in univariate analysis were further analyzed by multivariate analysis by using the Cox regression model, and HRs were calculated with 95% CI. A *p* value <0.05 was considered statistically significant. Survival curves and Time‐dependent ROC curves were plotted using RStudio (RStudio: Integrated Development for R. RStudio, Inc. URL http://www.rstudio.com/). All the curves were further optimized using Adobe Illustrator 2020 (Adobe Inc).

## 3 RESULTS

### 3.1 Demographics and clinicopathological characteristics

The present study included 358 patients with mid‐low rectal adenocarcinoma. A total of 238 patients (66.5%) were males and 120 were females (33.5%). The median age was 55 years (range from 24 to 81 years). Within a median interval of 7 weeks (range from 2 to 14) after NCRT completion, patients underwent surgery using the principles of total mesorectal excision (TME), including 147 open surgeries (41.1%) and 211 laparoscopic surgeries (58.9%). Low anterior resection (LAR) was performed in 174 patients (48.6%), abdominoperineal resection (APR) in 162 patients (45.3%), and the Hartmann procedure in 22 patients (6.1%). Two hundred and eighteen patients (60.9%) received adjuvant chemotherapy. According to the current AJCC 8^th^ TNM classification, 50 patients (14.0%) achieved pathological complete response (ypStage 0), 70 patients (19.5%) had ypStage I tumors, 113 patients (31.6%) had ypStage II tumors, and 125 patients (34.9%) had ypStage III tumors (including ypT0 N1‐2). The clinicopathological characteristics of the patients are shown in Table 1. M‐NLRG was associated with clinical N stage, adjuvant chemotherapy, TRG, M‐TTRG, and LRGmax.

**TABLE 1:** Clinical and pathological characteristics of patient treated with NCRT

	Total (%)	M‐NLRG−0 (%)	M‐NLRG−1 (%)	M‐NLRG−2 (%)	*p*
Age					0.236
≤60	244 (68.2)	153 (62.7)	60 (24.6)	31 (12.7)	
>60	114 (31.8)	80 (70.2)	19 (16.7)	15 (13.1)	
Gender					0.643
Male	238 (66.5)	152 (63.9)	56 (23.5)	30 (12.6)	
Female	120 (33.5)	81 (67.5)	23 (19.2)	16 (13.3)	
Distance from the anal verge					0.262
≤5 cm	252 (70.4)	165 (65.5)	51 (20.2)	36 (14.3)	
>5 cm	106 (29.6)	68 (64.2)	28 (26.4)	10 (9.4)	
Surgical Procedure					0.255
LAR	174 (48.6)	120 (69.0)	32 (18.4)	22 (12.6)	
APR	162 (45.3)	100 (61.7)	43 (26.6)	19 (11.7)	
Hartmann	22 (6.1)	13 (59.1)	4 (18.2)	5 (22.7)	
Clinical T stage					0.507
cT3	254 (70.9)	161 (63.4)	60 (23.6)	33 (13.0)	
cT4	104 (29.1)	72 (69.2)	19 (18.3)	13 (12.5)	
Clinical N stage					**0.002**
cN0	210 (58.7)	126 (60.0)	46 (21.9)	38 (18.1)	
cN+	148 (41.3)	107 (72.3)	33 (22.3)	8 (5.4)	
Interval completion of NCRT to surgery					0.633
≤7 weeks	204 (57.0)	137 (67.2)	42 (20.6)	25 (12.2)	
>7 weeks	154 (43.0)	96 (62.3)	37 (24.0)	21 (13.6)	
Adjuvant chemotherapy					**<0.001**
Yes	218 (60.9)	124 (56.9)	57 (26.1)	37 (17.0)	
No	140 (39.1)	109 (77.9)	22 (15.7)	9 (6.4)	
TRG					**<0.001**
TRG1	56 (15.6)	40 (89.3)	6 (10.7)	0 (0)	
TRG2	66 (18.4)	49 (74.2)	13 (19.7)	4 (6.1)	
TRG3	159 (44.4)	95 (59.7)	41 (25.8)	23 (14.5)	
TRG4	69 (19.3)	35 (50.7)	18 (26.1)	16 (23.2)	
TRG5	8 (2.3)	4 (50.0)	1 (12.5)	3 (37.5)	
M‐TTRG					**<0.001**
M‐TTRG 0	56 (15.6)	50 (89.3)	6 (10.7)	0 (0)	
M‐TTRG 1	114 (31.8)	84 (73.7)	25 (21.9)	5 (4.4)	
M‐TTRG 2	99 (27.7)	54 (54.5)	25 (25.3)	20 (20.2)	
M‐TTRG 3	74 (20.7)	38 (51.3)	19 (25.7)	17 (23.0)	
M‐TTRG 4	15 (4.2)	7 (46.6)	4 (26.7)	4 (26.7)	
LRGmax					**<0.001**
LRGmax 0	189 (52.8)	189 (100)	0	0	
LRGmax 1	44 (12.3)	44 (100)	0	0	
LRGmax 2	24 (6.7)	0	21 (87.5)	3 (12.5)	
LRGmax 3	60 (16.8)	0	45 (75.0)	15 (25.0)	
LRGmax 4	37 (10.3)	0	11 (29.7)	26 (70.3)	
LRGmax 5	4 (1.1)	0	2 (50.0)	2 (50.0)	

*p* value was calculated by *χ* ^2^ test.

Abbreviations: APR, abdominoperineal resection; LAR, low anterior resection; LRG, lymph node regression grade; LRGmax: maximum tumor regression grade in lymph node; M‐TTRG: modified ypT stage by combining ypT stage and TRG; NCRT, neoadjuvant chemoradiotherapy; TRG, tumor regression grade.

### 3.2 Lymph node evaluation

A total of 5106 LNs were harvested, including 425 PLNs (8.3%) and 4681 negative lymph nodes (NLNs, 91.7%). The median number of lymph nodes harvested from surgical resection specimens was 13 (range 2 to 49). The LRG distributions of each harvested LN were as follows, LRG0 in 4369 LNs (85.6%), LRG1 in 312 LNs (6.1%), LRG2 in 150 LNs (2.9%), LRG3 in 168 LNs (3.3%), LRG4 in 97 LNs (1.9%), and LRG5 in 10 LNs (0.2%) (Figure 1).

### 3.3 Prognostic value of current TNM staging system

**FIGURE 2:**
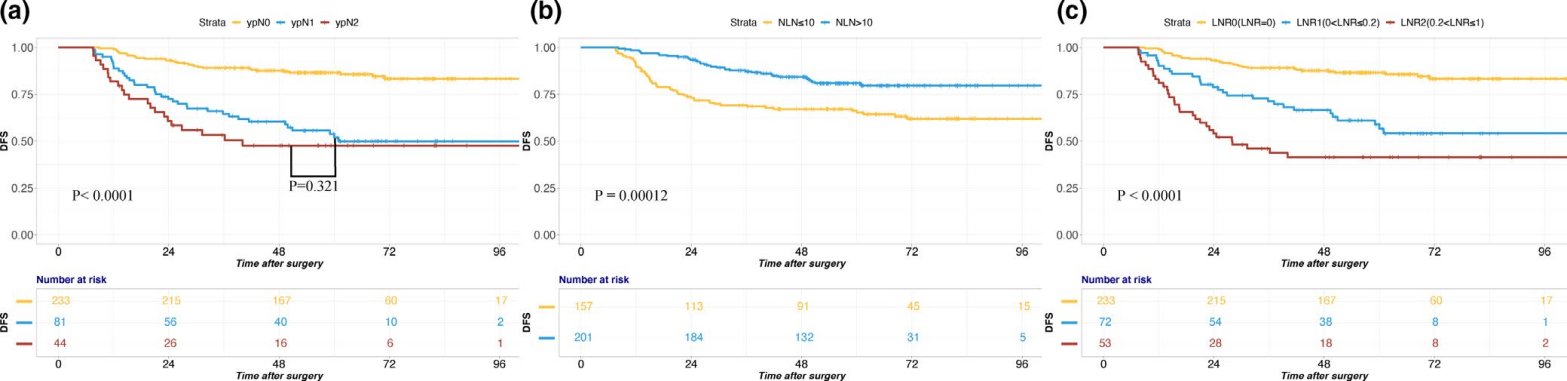
Disease‐free survival according to ypN, NLN, and LNR. A, No significant differences in 5‐year DFS were observed in patients with ypN1 and ypN2 according to the current TNM staging system (53.9% vs 47.7%, *p* = 0.321). B, Increased number of negative lymph nodes (NLN) was associated with improved survival. The 5‐year DFS of the two groups was 81.0% and 66.3%, respectively. C, Patients’ 5‐year DFS significantly decreased with increasing LNR. The 5‐year DFS rates of the three groups were 86.6%, 58.9%, and 41.5%, respectively

The DFS at 5 years according to the current AJCC TNM staging system is shown in Table 2. Among the ypN1‐2 subgroups, no significant differences in 5‐year DFS were observed in patients with ypN1 and ypN2 disease (53.9% vs 47.7%, *p* = 0.321, Table 2, Figure 2A). Then, we subdivided Stage III patients into three groups, and patients from stage IIIB had considerably better 5‐year survival outcomes than patients with stage IIIC theoretically. However, the 5‐year DFS of stage IIIB patients was even worse than that of stage IIIC patients. (46.2% vs 56.0%, Figure 4A).

**TABLE 2:** Association of pathological parameters with DFS

	N	5‐y DFS (%)	*p*
ypT stage			**<0.001**
ypT0	56	98.2	
ypT1	15	73.3	
ypT2	75	86.2	
ypT3	186	66.5	
ypT4	26	49.7	
ypN stage			**<0.001**
ypN0	233	86.6	
ypN1	81	53.9	
ypN2	44	47.7	
NLN			**<0.001**
>10	201	81.0	
≤10	157	66.3	
LNR			**<0.001**
LNR = 0	233	86.6	
0 < LNR ≤ 0.2	72	58.9	
0.2 < LNR ≤ 1	53	41.5	
TRG			**<0.001**
TRG1	56	98.2	
TRG2	66	87.1	
TRG3	159	71.3	
TRG4	69	57.9	
TRG5	8	0	
M‐TTRG			**<0.001**
M‐TTRG 0	56	98.2	
M‐TTRG 1	114	86.3	
M‐TTRG 2	99	68.3	
M‐TTRG 3	74	55.1	
M‐TTRG 4	15	33.3	
LRGmax			**<0.001**
LRGmax 0‐1	233	86.6	
LRGmax 2‐3	84	58.1	
LRGmax 4‐5	41	39.4	
LRGsum			**<0.001**
LRGsum = 0‐1	212	87.7	
LRGsum = 2‐12	108	61.4	
LRGsum >12	38	36.1	
LRG ratio			**<0.001**
≤1.4	236	86.8	
1.4‐3.5	86	54.6	
≥3.5	36	39.1	
M‐NLRG			**<0.001**
M‐NLRG 0	233	86.6	
M‐NLRG 1	79	60.3	
M‐NLRG 2	46	36.4	
TNM stage			**<0.001**
Stage 0	50	100	
Stage I	70	89.6	
Stage II	113	78.9	
Stage III	125	51.4	

*p* value was calculated by Kaplan–Meier survival analysis correlated with 5‐year DFS.

Abbreviations: LNR, lymph node ratio; LRG ratio, LRGsum was divided by the number of PLN; LRG, lymph node regression grade; LRGmax, maximum tumor regression grade in lymph node; LRGsum, sum score of LRG; M‐NLRG, modified ypN stage by combing positive lymph nodes, total number of retrieved lymph nodes and LRGmax; M‐TTRG, modified ypT stage by combining ypT stage and TRG; NLN, the number of negative lymph nodes; TRG, tumor regression grade.

### 3.4 Survival estimates according to LRGmax, LRGsum, and LRG ratio

**FIGURE 3:**
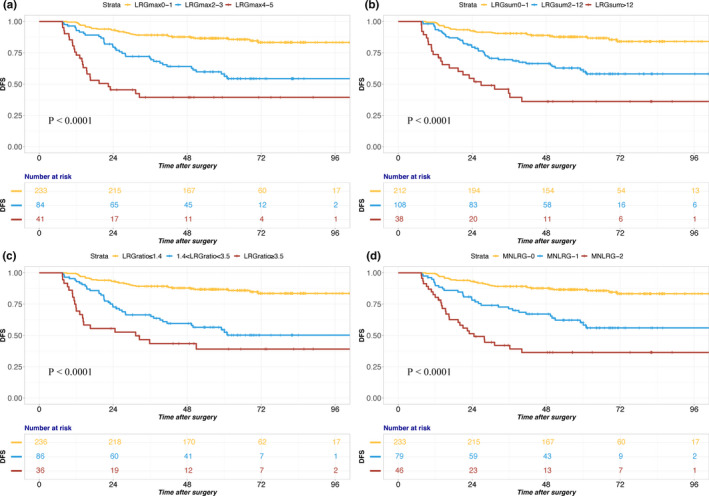
Disease‐free survival according to LRGmax, LRGsum, LRG ratio, and M‐NLRG. The DFS difference was highly significant between the three groups according to LRGmax (A), LRGsum (B), LRG ratio (C), and M‐NLRG (D).The X‐tile program was used to identify the optimal cut‐off value of LRGmax, LRGsum, LRG ratio, and M‐NLRG

The DFS difference was highly significant (*p* < 0.001), as were the differences between the groups of LRGmax 0–1 and LRGmax 2–3 (*p* < 0.001), the groups of LRGmax 0–1 and LRGmax 4–5 (*p* < 0.001) and the groups of LRGmax 2–3 and LRGmax 4–5 (*p* = 0.002, Table 2, Figure 3A). The LRGmax classification accommodates the group of patients treated with NCRT who were found to have no metastatic lymph nodes but had fibrotic lymph nodes in the specimen (N0, LRG1). The 5‐year DFS of the patients with true negative lymph nodes (LRG 0) was not significantly different from that of patients who had complete tumor response in the lymph nodes (LRG 1) (86.7% vs 86.1%, *p* = 0.822).

Additionally, our data indicated that a high LRGsum or LRG ratio (LRGsum divided by the number of PLN to obtain the average value of LRGsum) was significantly and negatively correlated with DFS (Table 2, Figure 3B,C). The DFS difference was highly significant in the subgroup analysis. The 5‐year DFS rates of the LRGsum 0–1, LRGsum 2–12, and LRGsum >12 groups were 87.7%, 61.4%, and 36.1%, respectively (Table 2, Figure 3B, *p *< 0.001). The same trend of 5‐year DFS was observed in the LRG ratio subgroup analysis. The 5‐year DFS was 86.8%, 54.6%, and 39.1% in the LRG ratio ≤1.4, 1.4 < LRG ratio <3.5 and LRG ratio ≥3.5 groups, respectively (Table 2, Figure 3C, *p*< 0.001).

These results suggested that lymph node regression grade was an important prognostic factor in rectal cancer patients with NCRT (Figure 3A‐C).

### 3.5 Prognostic value of the number of NLN and lymph node ratio (LNR)

An increased number of negative lymph nodes (NLN) was associated with improved survival in patients with NCRT. Patients with 10 or more negative lymph nodes had better 5‐year DFS than patients with 10 or fewer negative lymph nodes (81.0% vs 66.3%, *p* < 0.001, Table 2, Figure 2B).

Moreover, the lymph node ratio (LNR, number of positive LNs divided by the total number of retrieved LNs) was also associated with survival. Patients’ 5‐year DFS rates significantly decreased with increasing LNR values (*p* < 0.001, Table 2, Figure 2C). The 5‐year DFS rates in the LNR = 0, 0 < LNR ≤ 0.2, and 0.2 < LNR ≤ 1 groups were 86.6%, 58.9%, and 41.5%, respectively.

### 3.6 Combination of LRGmax and LNR into a new prognostic factor (M‐NLRG)

To incorporate ypN (the number of PLN), lymph node regression grade (LRGmax) and the number of NLN into one prognostic factor, LRGmax was multiplied by LNR to obtain a new parameter, M‐NLRG (modified ypN stage and lymph node regression grade). In the current TNM staging system, ypN stage is categorized by the absolute number of PLN into three categories. To create a comparable classification based on M‐NLRG, three risk categories were chosen for M‐NLRG as well: M‐NLRG 0 (M‐NLRG = 0), M‐NLRG 1 (M‐NLRG ≤ 1), and M‐NLRG 2 (M‐NLRG > 1). The estimated 5‐year DFS rates were 86.6%, 60.3%, and 36.4% for patients with M‐NLRG 0, M‐NLRG 1, and M‐NLRG 2, respectively (*p* < 0.001) (Table 2, Figure 3D).

### 3.7 Univariate and multivariate analyses of DFS

In univariate analysis, the factors associated with DFS were ypT stage, ypN stage, NLN, LNR, TRG, M‐TTRG, LRGmax, LRGsum, LRG ratio, M‐NLRG, and the current TNM staging system (Table 2).

A multivariate analysis including the factors of age, sex, ypT, ypN, M‐TTRG, and M‐NLRG showed that M‐TTRG (*p* < 0.001; HR 1.772; 95% CI 1.337–2.347) and M‐NLRG (*p* = 0.030; HR 1.915; 95% CI 1.064–3.448) were independent prognostic factors, but ypT (*p* = 0.764; HR 1.053; 95% CI 0.751–1.478) and ypN (*p* = 0.680; HR 1.138; 95% CI 0.616–2.103) were not (Table 3). The comparison between the ypT or ypN stage and the M‐TTRG or M‐NLRG classification suggested that the latter showed obvious advantages with higher hazard ratios and greater statistical significance.

**TABLE 3:** Multivariate analysis of DFS and pathological parameters

	HR	95% CI	*p*
ypT stage	1.053	0.751–1.478	0.764
ypN stage	1.138	0.616–2.103	0.680
M‐TTRG	1.772	1.337–2.347	**<0.001**
M‐NLRG	1.915	1.064–3.448	**0.030**

Abbreviations: CI, confidential interval; HR, hazard ratio; M‐NLRG, modified ypN stage by combing positive lymph nodes, total number of retrieved lymph nodes and LRGmax; M‐TTRG, modified ypT stage by combining ypT stage and TRG; TRG, tumor regression grade.

### 3.8 Proposed revision of the rectal cancer staging system to accommodate LRG and TRG

The current AJCC TNM staging system classifies patients without distant metastasis into different stages according to pT (tumor depth) and pN (nodal status). We propose a modified staging system by incorporating the tumor regression grade of the primary tumor and lymph nodes into the current TNM staging system for NCRT‐treated patients.

**FIGURE 4:**
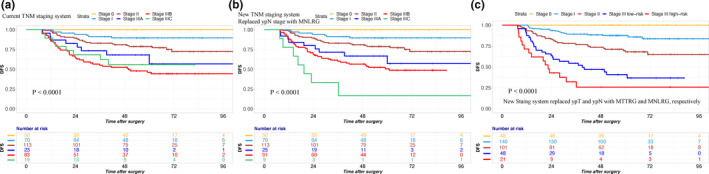
Disease‐free survival according to the current TNM staging system and modified TNM staging system. A, According to the survival curve based on the current TNM staging system, the 5‐year DFS of stage IIIB patients was even worse than that of stage IIIC patients (46.2% vs 56.0%). B, We replaced ypN stage with M‐NLRG, according to the modified staging system, and the survival curves of different tumor stages could clearly be distinguished from each other. C, We replaced the ypT stage with M‐TTRG and the ypN stage with M‐NLRG simultaneously to obtain a new staging system. We proposed combining two groups (M‐TTRG 0‐2 and M‐NLRG 1, M‐TTRG 3‐4 and M‐NLRG 0) into a new group because of similar 5‐year DFS rates (69.4% vs 69.6%). The remaining patients with lymph node metastases were divided into two groups: newStage III low‐risk (M‐TTRG 0‐2 and M‐NLRG 2, M‐TTRG 3‐4 and M‐NLRG 1) and newStage III high‐risk (M‐TTRG 3‐4 and M‐NLRG 2)

First, we replaced the ypN stage with M‐NLRG. More details on the 5‐year DFS rates of the current TNM staging system and the modified TNM staging system are provided in Table 4. According to the modified staging system, the survival curves of different tumor stages could clearly be distinguished from each other (Table 4 and Figure 4B).

**TABLE 4:** Proposed revision of modified ypTNM staging system combining with LRG

Prognostic Groups	ypT	ypN	5‐y DFS	ypT	M‐NLRG	5‐y DFS
Stage 0	T0	N0	100%	T0	M‐NLRG 0	100%
Stage I	T1‐2	N0	89.6%	T1‐2	M‐NLRG 0	89.6%
Stage II	T3‐4	N0	78.9%	T3‐4	M‐NLRG 0	78.9%
Stage IIIA	T0‐2	N1	68.2%	T0‐2	M‐NLRG 1	66.8%
	T1	N2a		T1	M‐NLRG 2a	
Stage IIIB	T3‐4	N1	46.2%	T3‐4	M‐NLRG 1	50.7%
	T2‐3	N2a		T2‐3 T1‐2	M‐NLRG 2a	
	T1‐2	N2b			M‐NLRG 2b	
Stage IIIC	T3	N2b	56.0%	T3	M‐NLRG 2b	16.7%
	T4	N2		T4	M‐NLRG 2	

M‐NLRG, modified ypN stage by combing positive lymph nodes, total number of retrieved lymph nodes and LRGmax.

Then, we replaced the ypT stage with M‐TTRG and the ypN stage with M‐NLRG simultaneously. Therefore, we obtained a new TNM staging system (Table 5 and Figure 4C). Patients with M‐TTRG 0‐2 and M‐NLRG 1 had similar 5‐year DFS rates compared to patients with M‐TTRG 3‐4 and M‐NLRG 0 (69.4% vs 69.6%). Therefore, we proposed combining those two groups into a new group (newStage II, Table 5 and Figure 4C). The remaining patients with lymph node metastases were divided into two groups: newStage III low‐risk (M‐TTRG 0‐2 and M‐NLRG 2, M‐TTRG 3‐4 and M‐NLRG 1) and newStage III high‐risk (M‐TTRG 3‐4 and M‐NLRG 2). More details on the 5‐year DFS rates of the current TNM staging system and the modified TNM staging system are provided in Tables 4 and 5.

**TABLE 5:** Proposed revision of modified ypTNM staging system combining with TRG and LRG

Prognostic Groups	M‐TTRG	M‐NLRG	5‐y DFS
newStage 0	M‐TTRG 0	M‐NLRG 0	100%
newStage I	M‐TTRG 1‐2	M‐NLRG 0	87.5%
newStage II	M‐TTRG 3‐4	M‐NLRG 0	69.6%
	M‐TTRG 0‐2	M‐NLRG 1	
newStage III low‐risk	M‐TTRG 0‐2	M‐NLRG 2	40.8%
	M‐TTRG 3‐4	M‐NLRG 1	
newStage III high‐risk	M‐TTRG 3‐4	M‐NLRG 2	25.7%

M‐NLRG, modified ypN stage by combing positive lymph nodes, total number of retrieved lymph nodes and LRGmax; M‐TTRG, modified ypT stage by combining ypT stage and TRG.

## 4 DISCUSSION

The current AJCC TNM staging system employs the same category definitions in patients with or without NCRT. We reported that the treatment response of primary tumors had prognostic significance and could refine the TNM staging system. In this study, we introduced the prognostic significance of lymph node regression grade (LRG) and for the first time we proposed a modified TNM staging system integrating LRG. The modified ypTNM staging system in combination with LNR and LRGmax could improve the DFS prediction in each subset of patients.

The evaluation of residual disease and histological regression after NCRT is important for assessing prognosis, identifying patients who benefitted most from the treatment, and selecting candidates for further adjuvant systemic therapy after surgery. We previously reported that the combination of ypT and the TRG of primary tumors could predict survival more precisely than ypT stage in patients with rectal cancer treated with NCRT.^8^ Currently, pathological assessment of the treatment response in primary tumor (TRG) after neoadjuvant therapy is routinely reported in pathological reports but not in lymph nodes (LRG). However, only the TRG of the primary tumor could not reflect the whole treatment response for an individual patient without LRG.

Distinguishing patients who never had nodal metastases from those who had lymph node disease successfully eliminated by neoadjuvant therapy may have prognostic value and provide therapeutic implications. Patients with lymph node involvement before neoadjuvant therapy may have never had lymph node involvement in their postoperative specimens.^17^ The total eradication of lymph node metastases was associated with better survival.^18^ This finding was shown by Rouzier et al., who reported 152 breast cancer patients with fine‐needle cytologically proven axillary LN metastases, and a better 5‐year DFS rate was observed in the group of patients with no involved nodes than in those with residual nodal disease at the time of surgery (73.5% vs 48.7%, *p* < 0.01).^19^ Given the downstaging effect, patients probably had a more advanced stage at diagnosis than that after operation. Subgroups of patients with different lymph node regression statuses may exhibit distinct outcomes.

In this study, we observed that LRGmax 0 patients had a slightly better outcome than LRGmax 1 patients, though both groups were ypN0. However, the prognosis did not differ significantly since patients who were lymph node negative had favorable outcomes. Longer follow‐up times and the enrollment of more patients are needed to examine the significance.

In rectal cancer without NCRT, pT was the key prognostic determination for patients without lymph node involvement. For NCRT‐treated rectal cancer, the patients with the best outcome were those patients with no lymph node metastases and no evidence of treatment response in lymph nodes. The 5‐year DFS rates of LRGmax 0–1, LRGmax 2–3, and LRGmax 4–5 were 86.6%, 58.1%, and 39.4%, respectively (*p* < 0.001). Compared with patients with advanced ypT stage, patients with early ypT stage had better outcomes, possibly because most patients also achieved a good response in the lymph nodes. Thus, we suggested a more thorough pathological assessment of the treatment effect on regional lymph nodes even though there were no residual tumor cells.

NCRT might not exert a uniform effect on all regional lymph node metastases. For patients with positive lymph nodes, each lymph node may present a different LRG after NCRT. Although the number of positive lymph nodes (PLN) is an independent prognostic factor for survival in patients with rectal cancer, our study revealed that there were no significant differences between the 5‐year DFS rates of ypN1 and ypN2 disease. Since lymph node metastasis is the most important prognostic factor and TRG in primary tumor was proven to be an independent prognostic factor by numerous studies, LRG may be an even more powerful prognostic factor than TRG in patients treated with NCRT.^19^ As proved in this study, both the M‐TTRG and M‐NLRG classifications were independent prognostic factors for DFS, but not ypT or ypN.

Different from the evaluation of TRG in primary tumors, even in an individual patient, tumor regression varied greatly among different lymph nodes, thus, it was critical to determine a suitable LRG score for evaluation. Mirbagheri demonstrated that the oncological outcome was better in patients with lymph node metastasis in whom a pathological response to preoperative treatment could be observed than that of the patients with no lymph node treatment response. In our study, the lymph node regression score, LRGmax, and LRGsum were significant predictors of disease outcome. However, for the LRGmax or LRGsum scoring system, the number of PLN, the most important prognostic factor in current TNM staging system, was not taken into account.

The current ypN stage in the AJCC TNM staging system is still based on the absolute number of PLN and was reported to be the most important prognostic factor after NCRT.^20^ However, considering the downstaging effect, the number of involved lymph nodes is low after preoperative chemoradiotherapy. The TNM staging system may not provide an accurate assessment of lymph node metastatic diseases because of stage migration.^21^ In this case, using the cutoff value of three PLNs to distinguish ypN1 and ypN2 was not suitable, and in this study, no significant differences in the 5‐year DFS rates were observed in patients with ypN1 and ypN2 disease (53.9% vs 47.7%, *p* = 0.321). Otherwise, only 34.9% of patients had lymph node metastasis after NCRT in this study. Furthermore, only 12.3% of patients were classified as ypN2 stage. Therefore, it is necessary to take LRG into consideration to modify the pathological TNM staging system in patients with NCRT.

In addition, some studies indicated that the number of negative lymph nodes also had significant prognostic value.^14^ In this study, an increased number of negative lymph nodes (NLN) was associated with improved survival.

To overcome the limitations of this metastatic lymph node number‐based nodal staging system, we introduced M‐NLRG as an alternative method. In an attempt to gain a relatively stable result while stratifying patients into prognostically meaningful groups, LRGmax was multiplied by LNR to obtain a new parameter, M‐NLRG (modified ypN stage and lymph node regression grade). The X‐tile program was used to identify the optimal cut‐off value of M‐NLRG based on DFS. As the ypN stage was categorized by the absolute number of PLN into three categories, we subdivided M‐NLRG into three risk categories. Theoretically, as a more ideal index that presents both the ratio of metastatic lymph nodes and the representative lymph node regression grade, M‐NLRG would better reflect the tumor burden in lymph nodes before and after NCRT.

Our study demonstrated that the combination of TRG and LRG with the current TNM staging system might be widely used in other types of cancers. However, there are inherent limitations to this single‐institution, retrospective study. Heterogeneity exists in the extent of preoperative workup, the neoadjuvant treatment regimens used, and the interval to surgery. In addition, although M‐NLRG can be considered a prognostic indicator, it still needs longer follow‐up in this cohort.

In conclusion, LRG is an important prognostic factor in rectal cancer patients treated with NCRT; the combination of LRG and TRG could refine the ypTNM rectal cancer staging system for cancer survival. However, further investigation of the value of our proposed TNM staging system in locally advanced rectal cancer treated with curative resection after neoadjuvant NCRT is needed.

## CONFLICTS OF INTEREST

The authors declare that they have no competing interests.

## ETHICAL STATEMENT

This study was approved by Institutional Review Board of the Cancer Hospital, Chinese Academy of Medical Sciences (CHCAMS).

5

**FIGURE 5:**
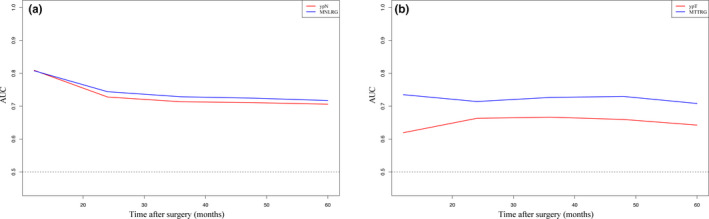
The time‐dependent ROC curve of ypN, M‐NLRG, ypT, and M‐TTRG. M‐NLRG (A) and M‐TTRG (B) had better prognosis predictive value compared to ypN and ypT stage, respectively
